# Revista Medico—Quirurgica Y Dentistica

**Published:** 1868-08

**Authors:** 


### BIBLIOGRAPHICAL NOTICE.

*Revista Medico-- Quirurgica Y Dentistica*, De Los Sres.—Wilson Y Gonzalez, Habana. This is the title of a new Spanish Journal of Medicine, Surgery and Dentistry, published in Havana, Cuba.

We believe this is the first attempt made to establish a Journal of this kind in the West Indies, and from the well-known ability of Dr. Erastus Wilson, one of its editors, we are confident that it will meet with encouragement from the Spanish portion of the profession. The two numbers received, April and July, are handsomely gotten up, and well supplied with original articles and selected matter. We welcome this new candidate for professional honors and patronage most cordially, and wish it abundant success. It is published quarterly, each number containing over one hundred pages.

Dr. John Howe, 311 W. Seventeenth Street, New York, is the agent for this country.

